# The association between early maladaptive schemas and romantic relationship satisfaction

**DOI:** 10.3389/fpsyg.2024.1460723

**Published:** 2024-12-19

**Authors:** Lili Kover, Gergo Jozsef Szollosi, Ede Frecska, Antal Bugan, Roland Berecz, Aniko Egerhazi

**Affiliations:** ^1^Clinical Psychology Center, University of Debrecen Medical Center, Debrecen, Hungary; ^2^Department of Psychiatry, University of Debrecen Medical Center, Debrecen, Hungary; ^3^Faculty of Economics and Business Coordination Center for Research in Social Sciences, University of Debrecen, Debrecen, Hungary

**Keywords:** relationship satisfaction, early maladaptive schemas, actor-partner interdependence model, sex differences, romantic partner

## Abstract

**Introduction:**

Relationship dynamics could be shaped by schemas, and relationship satisfaction could be depend on how couples perceive each other through these schemas. The main aim of this study was to assess how early maladaptive schemas are related to relationship satisfaction for both males and females in a relationship, by highlighting factors that contribute to relationship satisfaction, with a particular focus on the interaction of schemas between males and females.

**Methods:**

The study involved a total of healthy 47 different-gender couples. Participants completed the Personal Assessment of Intimacy in Relationship (PAIR) and Young’s Schema questionnaires. Actor-partner interdependence models (APIM) were created to assess which factors contributed to satisfaction.

**Results:**

Our study identifies sex differences in early maladaptive schemas, revealing complex interactions within romantic relationships. Strong associations were observed between both sexes’ maladaptive schemas, suggesting mutual influence. The emotional deprivation schema is associated with reduced satisfaction for both partners. For females, the abandonment schema is linked to decreased both their own and their partner’s satisfaction, while the mistrust/abuse schema is related to lower satisfaction in the male partner. Social isolation and defectiveness/shame schemas are associated with lower female satisfaction but do not appear to affect their partners, whereas male satisfaction is reduced by vulnerability to harm. Schemas such as failure to achieve, dependence/incompetence and enmeshment show little association with relationship satisfaction. Approval-seeking schema is linked to reduced female satisfaction, and negativity/pessimism is associated with reduced male satisfaction.

**Discussion:**

Our research provides a nuanced insight into the interactions between partners’ schemas, thus improving the understanding of how maladaptive schemas may contribute to relationship outcomes.

## 1 Introduction

The examination of factors influencing romantic relationship satisfaction has been an ongoing pursuit, with existing definitions encompassing notions of conformity, happiness, success, and quality (Fincham et al., 1997).

Researchers emphasize the complexity of relationship satisfaction, with various dimensions such as intimacy, communication, and shared values playing crucial roles. While these dimensions have been well studied, less attention has been paid to how other internal psychological elements, particularly early maladaptive schemas, shape relationship satisfaction. Early studies, such as Bernard’s (1976) suggested that partners often perceive their relationship differently. This emphasizes the particular need to explore, beyond external determinants such as gender roles, the internal cognitive structures that shape these perceptions. While more traditional explanations such as socialisation and gender roles (Sakalli-Ugurlu, 2003; Eagly and Wood, 1999) provide insight into the development of these perceptions, they cannot explain the deeper patterns, such as early maladaptive schemas, that influence how individuals interpret relationship dynamics.

Rooted in temperament and environmental influences, early maladaptive schemas occur as deep personality patterns, shaping individuals’ interpretations of their partners’ behavior within relationships (Young et al., 2003). According to the schema theory, throughout the development, five core emotional needs should be fulfilled: secure attachment to others, autonomy, competence, and a sense of identity, realistic limits and self-control, freedom to express valid needs and emotions, and spontaneity and play. Failure to meet these needs results in the formation of early maladaptive schemas in various fields: (1) Disconnection/Rejection; (2) Impaired Autonomy and/or Performance; (3) Impaired Limits; (4) Other-Directedness; (5) Overvigilance/Inhibition (Young et al., 2003) (see Table 1). Young and Gluhoski (1997) identified 18 schemas which have an effect on relationships (we will use the terms “schema” and “early maladaptive schema” virtually interchangeably). These schemas influence how individuals interpret their partner’s behavior, often unconsciously shaping interactions and perceptions in ways that could potentially reduce satisfaction.

**Table 1:** Early maladaptive schemas and schema domains.

Disconnection and rejection	Impaired autonomy and performance	Impaired limits	Other-directedness	Overvigilance and inhibition
Emotional deprivation (ED)	Dependence/incompetence (DI)	Entitlement/grandiosity (ET)	Subjugation (SB)	Negativity/pessimism (NP)
Abandonment/instability (AB)	Vulnerability to harm or illness (VH)	Insufficient self-control/self-discipline (IS)	Self-sacrifice (SS)	Overcontrol/emotional inhibition (EI)
Mistrust/abuse (MA)	Enmeshment/underdeveloped self (EM)		Approval-seeking/recognition-seeking (AS)	Unrelenting standards/hypercriticalness (US)
Defectiveness/shame (DS)	Failure to achieve (FA)			Punitiveness (PU)
Social Isolation (SI)				

Although schemas play a crucial role in shaping relationship dynamics, empirical studies specifically examining their impact on relationship satisfaction are still relatively rare. Notably, studies, including those by Dumitrescu and Rusu (2012), Eftekhari et al. (2018), and Zafary and Mohammadzadeh (2015), have demonstrated the negative relationship between early maladaptive schemas and relationship satisfaction. In addition, specific schema, such as emotional deprivation, can also predict relationship dissatisfaction (Esmaili et al., 2016). Furthermore subjugation and self-sacrifice schemas have been linked to dissatisfaction, with some schemas showing gender specificity (Welburn et al., 2002). However, these studies have not adequately explored how schemas influence interactions between partners in a relationship, particularly in terms of gender differences.

Relationship satisfaction is the measure of how needs, expectations and desires are realized in romantic relationships (Rusbult, 1983) Cognitive attributions, rooted in individual vulnerability (fundamentally negative self-concepts, early maladaptive schemas) and maladaptive coping strategies, correlate with relationship satisfaction (McNulty and Karney, 2004). Thus, understanding how partners internally perceive and interpret relationship events becomes central to understanding satisfaction dynamics. Related factors such as commitment, time spent together, perceived relationship alternatives, and long-term goals also support the idea that cognitive schemas play a role in shaping relationship satisfaction (Bradbury et al., 2000; Stanley et al., 2002). Furthermore, cognitive schemas from intimate experiences, as described by Franiuk et al. (2004), further emphasize how deep-rooted cognitive structures influence romantic interactions.

We hypothesise that early maladaptive schemas might impact relationship satisfaction among romantic partners, with distinct patterns and interactions between male and female partners, potentially shaping overall relationship dynamics. By providing a deeper insight into how maladaptive schemas affect relationship satisfaction, this study has the potential to significantly improve relationship counselling and therapy. Identification of the most important distortions in both male and female cognitions could allow therapists to develop more targeted interventions to address these issues early on, thereby improving long-term relationship stability. Our findings could also contribute to relationship education programmes and self-help resources, helping individuals to better understand and manage their schemas. In addition, the findings could have wider public health benefits by reducing mental health problems associated with relationship dissatisfaction, such as anxiety and depression. The study may also inform gender-sensitive therapeutic interventions, helping to target treatments more effectively for males and females.

## 2 Materials and methods

### 2.1 Subjects

Healthy volunteers were recruited from the staff of the Department of Psychiatry of the Medical Faculty of the University of Debrecen and their acquaintances. The data collection period lasted for one year, until 2023. The study couples were formed by the participants together with their partners. Inclusion criteria were that participants had to be over 18 years of age and part of a mixed-sex couple that had been in a relationship for at least six months. Only cisgender individuals were included, as one of the aims of the study was to assess the role of gender in the association between relationship satisfaction and early maladaptive schemas. Participants were informed of the aims and protocol of the study and gave their consent to participate. The research protocol was approved by the local ethics committee.

The questionnaires were sent to the volunteer couples, who were asked to complete them independently and without supervision. Instructions emphasised that participants should complete the questionnaire alone and not discuss their answers with their partners. All responses were returned anonymously and the questionnaires were finally received from 256 couples; 162 couples were excluded, because in 93 cases questionnaires were not filled out by both partners of the couple and 69 included incomplete data. The questionnaires with missing data were not taken into consideration during the data analysis.

The final sample consisted of 94 individuals (47 couples, with 47 females and 47 males) with a mean age of 50.94 years (SD = 6.4). The female mean age was 51.3 (SD = 6.2), and the male mean age, 49.7 (SD = 6.1). The couples had been in a relationship for an average of 24.1 (SD = 6.9) months.

### 2.2 Methods

A complex questionnaire was administered, but given the focus of the current study, only the relevant parts of the questionnaire are described in detail here. At the beginning of the questionnaire, participants were asked to provide some demographic information, such as gender, age, highest educational level and length of their current relationship.

#### 2.2.1 Young schema questionnaire

The Young Schema Questionnaire (YSQ, Young and Brown, 2001) was used to uncover the early maladaptive schemas of the couples. The YSQ is a measure of early maladaptive schemas that are responsible for the development and maintenance of pathological personality traits and includes 18 maladaptive schemas and the social undesirability schema. The questionnaire is a self-report, paper and pencil test containing a total of 244 items.

The respondent rates the items on a six-point Likert scale (endpoints: 1-not at all typical, 6-perfectly typical), depending on how typical the given statement is of him or her. For example, one item of the questionnaire is “*Most of the time, there was nobody who would have really listened and understood me, realising my true needs and emotions*.” The questionnaire was translated into Hungarian by Unoka et al. (2004). They confirmed both the discriminant and convergent validity of the Hungarian version. In addition to confirming the internal consistency of the questionnaire, the discriminant validity of the Hungarian version has been demonstrated in the study of patients with eating disorders (Unoka et al., 2007).

#### 2.2.2 Personal assessment of intimacy in relationship

The Personal Assessment of Intimacy in Relationship (PAIR) questionnaire, developed by Schaefer and Olson (1981), was designed to assess relationship satisfaction from an intimate relationship perspective. The PAIR inventory measures intimacy across multiple dimensions, including emotional, social, sexual, intellectual and recreational intimacy. Examples of items include: emotional – “*I often feel distant from my partner”*; sexual – “*Sexual expression is an essential part of our relationship.*”; intellectual – *“My partner frequently tries to change my ideas”*; and recreational – *“We enjoy the same recreational activities.”* The final version of the questionnaire includes four subscales and 24 items, with Cronbach’s alpha values between 0.70 and 0.77, indicating good reliability (Sabatelli, 1988).

### 2.3 Statistical analysis

Continuous data are presented with means and standard deviations, although the data were analysed using non-parametric tests, as the vast majority of variables did not follow a normal distribution when testing the normality of the data with Shapiro–Wilk tests, and therefore the matched means were analysed using Wilcoxon matched-pairs signed-rank tests. The internal consistency of the schema variables was assessed separately for males, females, and the combined sample. The effect and interactions of the scores of the different schemes were analysed using Spearman correlations.

Finally, mixed models were used as actor-partner independence models (APIM) to see exactly how each schema affects the relationship satisfaction of each member of each pair, potentially adjusted for the interactions regarding the schemas of the participants. The results were considered significant if the *p*-values of the given statistical tests were below 0.05. All data analysis was done using IBM SPSS Statistics (Version 27) software.

## 3 Results

### 3.1 Results on early maladaptive schemas for males and females

For females the schema variables Cronbach’s alpha was 0.912, which indicates that the schema items are strongly interrelated and consistently measure the underlying construct related to maladaptive schemas among females. For males the schema variables also showed strong internal consistency, with a Cronbach’s alpha of 0.890. When the male and female schema variables were combined, the an overall Cronbach’s alpha was equal to 0.934, reflecting excellent internal consistency across the total sample.

Males scored a mean of 16.6 (8.06) on the emotional deprivation (ed) schema, significantly higher than females’ mean of 14.07 (5.65), with a *p*-value of 0.010 (see Table 2). The mean score for males on the social isolation (si) schema was 15.48 (6.19), significantly higher than the mean score for females of 14.63 (7.17), with a *p*-value of 0.039. Males had an average score of 21.89 (7.17) on the defectiveness/shame (ds) schema. This was significantly higher than females’ mean of 19.65 (4.58), with a *p*-value of 0.032. Males’ average score on the self-sacrifice (ss) schema was 50.49 (14.21), significantly lower than females’ average of 57.17 (16.17), with a *p* value of 0.001. Males scored an average of 23.33 (9.52) on the negativity/pessimism (np) schema. This was significantly higher than the female average of 24.21 (8.91), with a *p*-value of 0.019. Regarding the other schemas’ central values no significant differences were observed between males and females.

**Table 2:** Descriptive statistics of the schemas regarding males and females.

Schemas	Male		Female		*p*-value
	Mean	SD	Mean	SD	
Emotional deprivation	16.60	8.06	14.07	5.65	0.010*
Abandonment	33.81	12.07	31.51	10.18	0.910
Mistrust/abuse	37.74	13.10	35.15	12.27	0.119
Social isolation	15.48	6.19	14.63	7.17	0.039*
Defectiveness/shame	21.89	7.17	19.65	4.58	0.032*
Failure to achieve	13.35	4.94	14.08	5.22	0.328
Dependence/incompetence	22.70	7.56	22.81	6.36	0.120
Vulnerability to harm or illness	24.36	8.87	24.58	7.47	0.302
Enmeshment/undeveloped self	15.17	4.65	16.60	5.98	0.268
Subjugation	17.17	6.13	17.50	5.34	0.160
Self-sacrifice	50.49	14.21	57.17	16.17	0.001*
Emotional inhibition	16.60	6.48	15.13	5.87	0.477
Unrelenting Standards/hypercriticalness	45.50	16.07	45.32	13.76	0.500
Entitlement/grandiosity	24.86	8.72	23.92	8.03	0.165
Insufficient self-control/self-discipline	30.04	11.73	29.55	9.67	0.822
Approval-seeking/recognition-seeking	27.33	9.18	28.61	10.32	0.213
Negativity/pessimism	23.33	9.52	24.21	8.91	0.019*
Punitiveness	38.46	13.48	35.94	12.04	0.500

Significant findings are highlighted with “*,” significance level was 0.05.

### 3.2 Correlation between males’ and females’ early maladaptive schemas

There were several significant correlations between males’ and females’ schema scores (see Table 3). Emotional deprivation in males correlated (rho = 0.46, *p* < 0.001) with vulnerability to harm or illness (vh) in females. Both sexes showed strong correlations within schemas for abandonment (ab) (rho = 0.45, *p* < 0.001), mistrust/abuse (ma) (rho = 0.58, *p* < 0.001), dependence/incompetence (di) (rho = 0.55, *p* < 0.001), enmeshment/underdeveloped self (em) (rho = 0.47, *p* < 0.001), subjugation (sb) (rho = 0.49, *p* < 0.001), self-sacrifice (rho = 0.43, *p* < 0.001), insufficient self-control/self-discipline (is) (rho = 0.54, *p* < 0.001), approval-seeking/recognition-seeking (as) (rho = 0.61, *p* < 0.001), and negativity/pessimism (rho = 0.61, *p* < 0.001) Social isolation in males correlated (rho = 0.44, *p* < 0.001) with negativity/pessimism in females, while defectiveness/shame in males correlated (rho = 0.51, *p* < 0.001) with dependence/incompetence in females. Males’ vulnerability to harm or illness schema scores were most highly correlated (rho = 0.43, *p* < 0.001) with females’ vulnerability to harm or illness, enmeshment/underdeveloped self (rho = 0.43, *p* < 0.001), and subjugation schemas (rho = 0.43, *p* < 0.001). Males’ emotional inhibition/overcontrol (ei) correlated (rho = 0.52, *p* < 0.001) with females’ defectiveness/shame, and males’ unrelenting standards/hypercriticism (us) correlated (rho = 0.46, *p* < 0.001) with females’ negativity/pessimism. Finally, males’ punitiveness (pu) correlated (rho = 0.46, *p* < 0.001) with females’ enmeshment/underdeveloped self.

**Table 3:** Correlation coefficients of schema among males and females.

		Male																	
		ed	ab	ma	si	ds	fa	di	vh	em	sb	ss	ei	us	et	is	as	np	pu
Female	ed	0.46*	0.28*	0.39*	0.22*	0.35*	0.31*	0.29*	0.35*	0.24*	0.39*	0.17	0.49*	0.21	0.31*	0.32*	0.47*	0.36*	0.27*
	ab	0.39*	0.45*	0.42*	0.26*	0.29*	0.11	0.09	0.22*	0.08	0.11	0.20	0.34*	0.34*	0.46*	0.30*	0.37*	0.37*	0.31*
	ma	0.29*	0.34*	0.58*	0.33*	0.20	0.14	0.10	0.28*	0.18	0.20	0.19	0.33*	0.32*	0.51*	0.31*	0.48*	0.35*	0.40*
	si	0.26*	0.28*	0.28*	0.37*	0.38*	0.33*	0.30*	0.12	0.24*	0.25*	0.01	0.39*	0.12	0.24*	0.22*	0.26*	0.09	0.01
	ds	0.37*	0.29*	0.46*	0.34*	0.47*	0.39*	0.51*	0.35*	0.42*	0.4*	0.06	0.52*	0.19	0.28*	0.43*	0.29*	0.22*	0.15
	fa	0.31*	0.25*	0.34*	0.37*	0.44*	0.43*	0.40*	0.29*	0.36*	0.28*	0.08	0.46*	0.17	0.24*	0.41*	0.40*	0.27*	0.22*
	di	0.3*	0.26*	0.40*	0.38*	0.51*	0.44*	0.55*	0.27*	0.42*	0.37*	0.05	0.51*	0.13	0.28*	0.32*	0.38*	0.22	0.11
	vh	0.48*	0.43*	0.48*	0.37*	0.42*	0.39*	0.47*	0.43*	0.44*	0.45*	0.17	0.5*	0.32*	0.33*	0.37*	0.33*	0.34*	0.27*
	em	0.42*	0.33*	0.40*	0.39*	0.24*	0.27*	0.35*	0.43*	0.47*	0.23*	0.22*	0.38*	0.36*	0.30*	0.37*	0.40*	0.41*	0.46*
	sb	0.23*	0.29*	0.34*	0.38*	0.37*	0.41*	0.40*	0.43*	0.39*	0.49*	0.27*	0.46*	0.38*	0.34*	0.37*	0.45*	0.44*	0.33*
	ss	0.19	0.18	0.16	0.10	0.17	0.07	0.14	0.24*	0.05	0.26*	0.43*	0.20	0.33*	0.23*	0.19	0.32*	0.37*	0.34*
	ei	0.25*	0.29*	0.36*	0.31*	0.36*	0.34*	0.35*	0.36*	0.32*	0.27*	0.04	0.44*	0.24*	0.26*	0.33*	0.31*	0.21	0.19
	us	0.19	0.27*	0.16	0.27*	0.17	0.10	0.08	0.10	0.11	0.08	0.22*	0.19	0.37*	0.18	0.19	0.30*	0.19	0.13
	et	0.20	0.26*	0.34*	0.28*	0.09	0.11	0.01	0.25*	0.15	0.14	0.24*	0.19	0.21	0.41*	0.25*	0.34*	0.26*	0.27*
	is	0.33*	0.31*	0.36*	0.36*	0.17	0.25*	0.21	0.40*	0.20	0.17	0.13	0.32*	0.30*	0.38*	0.54*	0.38*	0.40*	0.44*
	as	0.37*	0.25*	0.45*	0.35*	0.20	0.37*	0.22*	0.26*	0.23*	0.28*	0.35*	0.52*	0.40*	0.41*	0.32*	0.61*	0.38*	0.44*
	np	0.39*	0.41*	0.36*	0.44*	0.26*	0.35*	0.25*	0.29*	0.25*	0.30*	0.29*	0.51*	0.46*	0.51*	0.37*	0.43*	0.61*	0.40*
	pu	0.17	0.25*	0.14	0.12	0.14	0.19	0.01	0.16	0.10	0.02	0.17	0.16	0.33*	0.22*	0.14	0.37*	0.27*	0.37*

Significant findings are highlighted with “*,” significance level was 0.05.

Emotional deprivation in females correlated (rho = 0.47, *p* < 0.001) with approval seeking in males, and social isolation in females correlated (rho = 0.39, *p* < 0.001) with emotional inhibition/overcontrol in males. Female failure to achieve (fa) correlated (rho = 0.46, *p* < 0.001) with male emotional inhibition/overcontrol, and female dependence/incompetence had the strongest correlation (rho = 0.51, *p* < 0.001) with male defectiveness/shame and emotional inhibition/overcontrol (rho = 0.51, *p* < 0.001). Vulnerability to harm or illness in females correlated (rho = 0.48, *p* < 0.001) with emotional deprivation and mistrust/abuse in males (rho = 0.48, *p* < 0.001), while subjugation in females correlated with approval-seeking/recognition-seeking (rho = 0.45, *p* < 0.001) in males. Finally, females’ punishment schema correlated (rho = 0.37, *p* < 0.001) significantly with males’ approval-seeking/recognition-seeking and punishment (rho = 0.37, *p* < 0.001) schemas.

### 3.3 Actor-partner interdependence model results

The emotional deprivation schema within the disconnection and rejection domain was an imperative significant factor associated with relationship satisfaction for both males and females. Consequently, higher levels of emotional deprivation were related with reduced satisfaction for both partners, suggesting that emotional deprivation might has a mutual negative effect on relationship quality in couples (see Table 4). The abandonment/instability schema showed no effect on own relationship satisfaction, but it significantly reduced both partner’s satisfaction. The mistrust/abuse schema showed partner effect: while it did not influence satisfaction directly, it was associated with lower satisfaction in male partners. The social isolation schema had no effect on males’ satisfaction, but for females, it was associated with lower partner satisfaction.

**Table 4:** APIM results, schema interactions between males and females.

		Effect: actor	Effect: partner	Effect: actor	Effect: partner
		Coeff.	*p*-value	Coeff.	*p*-value
ED	Male	−0.062	0.004*	−0.062	0.042*
	Female	−0.067	0.006*	−0.091	0.008*
AB	Male	−0.024	0.103	−0.032	0.075
	Female	−0.032	0.047*	−0.057	0.004*
MA	Male	−0.011	0.47	−0.029	0.089
	Female	−0.005	0.795	−0.045	0.019*
SI	Male	−0.044	0.124	0.011	0.668
	Female	−0.068	0.035*	−0.019	0.495
DS	Male	−0.036	0.179	0.027	0.508
	Female	−0.073	0.016*	0.010	0.825
FA	Male	−0.010	0.794	0.008	0.83
	Female	−0.007	0.871	−0.073	0.065
DI	Male	−0.003	0.904	0.002	0.942
	Female	0.002	0.950	−0.062	0.086
VH	Male	−0.050	0.017*	0.005	0.853
	Female	−0.019	0.417	−0.040	0.154
EM	Male	−0.006	0.875	0.004	0.905
	Female	−0.040	0.377	0.007	0.844
SB	Male	−0.071	0.025*	0.012	0.745
	Female	−0.102	0.004*	−0.008	0.847
SS	Male	−0.019	0.160	−0.004	0.758
	Female	−0.023	0.123	−0.017	0.195
AS	Male	−0.061	0.008*	0.032	0.116
	Female	−0.067	0.01*	0.006	0.786
EI	Male	−0.058	0.058	0.014	0.679
	Female	−0.105	0.002*	0.028	0.441
US	Male	−0.012	0.303	−0.008	0.585
	Female	−0.015	0.257	−0.034	0.025*
NP	Male	−0.077	<0.001*	0.029	0.181
	Female	−0.044	0.072	−0.014	0.577
PU	Male	−0.030	0.030*	0.003	0.822
	Female	−0.019	0.215	−0.017	0.305
ET	Male	−0.042	0.051	−0.006	0.785
	Female	−0.051	0.036	−0.026	0.320
IS	Male	−0.022	0.180	0.004	0.828
	Female	−0.050	0.007*	0.022	0.327

Significant findings are highlighted with “*,” significance level was 0.05.

The defectiveness/shame schema had no effect on males’ satisfaction or their partners’, but for females, it might reduce their own satisfaction without affecting their partner’s. The failure to achieve and dependence/incompetence schemas showed no statistically significant impact on relationship satisfaction. The vulnerability to harm or illness schema was negatively correlating with males’ own relationship satisfaction but had no effect on their partner’s. For females, this schema did not affect either their own or their partner’s satisfaction. No significant association was found between the enmeshment/underdeveloped self schema and relationship satisfaction for either males or females. The subjugation schema significantly reduced the partner’s satisfaction, but had no significant effect on the males or females own satisfaction. No significant effects on relationship satisfaction were found for the self-sacrifice schema. The approval-seeking schema was negatively related to both males’ and females’ own satisfaction but did not significantly impact their partners’ satisfaction.

The emotional inhibition schema had no effect on males’ satisfaction, but for females, it was significantly associated with a worsened self relationship satisfaction without affecting their partners. The unrelenting standards schema significantly affected females, might leading to a negative impact on their male partners’ satisfaction. The negativity/pessimism and punitiveness schemas significantly affected only males, reducing their own satisfaction. The entitlement/grandiosity schema within the impaired limits schema domain showed no significant impact on relationship satisfaction for either males or females. The insufficient self-control schema significantly reduced satisfaction for females but had no effect on their partners’ satisfaction.

## 4 Discussion

This study aimed to investigate the relationship between early maladaptive schemas and relationship satisfaction across sexes, since we hypothesised that early maladaptive schemas might be associated with satisfaction. We studied a heterogeneous sample, examining mixed-sex couples where both members were asked to score their early maladaptive schemas and relationship satisfaction. Our findings are consistent with previous studies (Celsi et al., 2021; Bal, 2023; Bishop et al., 2021) suggesting that males score higher on emotional deprivation, social isolation and defectiveness/shame schemas. They may feel more emotionally unsupported in relationships, possibly due to social norms that discourage male vulnerability. Perhaps influenced by societal pressures for independence and emotional expression, they may feel more detached. These schemas are associated with feelings of loneliness and lower relationship satisfaction (Astaneh et al., 2013). Females, on the other hand, scored higher on the self-sacrifice schema, which may reflect traditional gender roles that encourage prioritising the needs of others, which can lead to imbalance and stress in relationships (Shorey et al., 2012a, 2012b; Singh et al., 2022). This may be due to traditional gender roles that emphasize the responsibility of females to support and care for others. While the self-sacrifice schema can be linked to creating harmony in relationships, it can also lead to distress or burnout, especially when the dynamic becomes one-sided. Furthermore, females scored higher on the negativity/pessimism schema, indicating a tendency towards a more pessimistic attitude, which may influence their relationship dynamics (Shorey et al., 2012a, 2012b).

### 4.1 Similar and complementer schemas in males and females

Our study shows strong relationships between different maladaptive schemas in males and females, suggesting complex interactions in romantic relationships. The difference in the prevalence of maladaptive schemas between males and females has been the subject of much research, but the complementarity of patterns within a relationship has been little studied. According to our findings, males’ emotional deprivation schema is highly associated with females’ vulnerability to harm or illness schema. This is consistent with the results of Celsi et al. (2021). Our findings may suggest that males perceive their partners as emotionally unavailable, and thus their unmet emotional needs may be linked to heightened their partners’ fears for their own personal safety and happiness. The associations between different schemas in both sexes suggest the interdependent nature of maladaptive schemas in romantic relationships, creating mutually reinforcing cycles. According to attachment theory (Bowlby, 1980), this dynamic may reflect the triggering of anxious attachment in women, where emotional deprivation in their partner may heighten fears of abandonment. In males, on the other hand, a more avoidant attachment style may be activated, where emotional closeness is perceived as a threat, triggering further emotional withdrawal. This interplay between anxious and avoidant tendencies could create a self-reinforcing cycle of emotional distance, with each partner strengthening the other’s maladaptive schema.

Both sexes are strongly related within schemas for abandonment, mistrust/abuse, dependence/incompetence, enmeshment/underdeveloped self, subjugation, self-sacrifice, insufficient self-control, approval-seeking, negativity/pessimism. For example, male scores on the abandonment schema are closely related to female scores on the same schema, suggesting a shared fear of being left alone. In the same way, high correspondence reinforces the mistrust/abuse schema often due to shared past trauma (Estévez et al., 2016).

Complementarity shows how the maladaptive schemas of one partner may inform and reinforce the different but related schemas of the other one. For illustration, males with social isolation schema may enhance females’ negativity/pessimism schema, thus producing a cycle of detachment. This could mean that the more isolated the male partner feels, the more pessimistic the female partner is about the relationship. This relationship is supported by research showing that social isolation schemas are associated with negative social self-perceptions and loneliness over time (Roelofs et al., 2011).

In addition, the defectiveness or shame schema in males is linked to a greater dependence and incompetence schema in females, reinforcing a dynamic of dependency. In psychodynamic terms, this pattern could be interpreted as a manifestation of codependency, where one partner’s low self-esteem (defectiveness/shame) is related to the other partner’s need for validation and care (dependence/incompetence). Eventually, these reinforcing patterns may inhibit the emotional growth of both partners and contribute to a stagnant, unhealthy relationship. Males’ vulnerability to harm or illness schema scores were most strongly related to females’ vulnerability to harm or illness, enmeshment/underdeveloped self, and subjugation schemas. Similar to our findings, Shorey et al. (2012a, 2012b) provide valuable insights into the relationship between early maladaptive schemas and differences between the sexes. Male emotional inhibition/overcontrol related to female defectiveness/shame, and male unrelenting standards/hypercriticism corresponded to female negativity/pessimism, a finding consistent with Santos et al.'s (2018) findings. Finally, male punitiveness correlated with female enmeshment/underdeveloped self. By identifying the complementary schemas, it can be seen how the partners’ different schemas interact and influence each other, creating specific relationship dynamics.

Emotional deprivation in females associated with approval seeking in males, and social isolation in females related to emotional inhibition/overcontrol in males. This is consistant with the findings of Celsi et al. (2021) and Roelofs et al. (2011). This result could imply that when males are emotionally withdrawn, this contributes to females feeling isolated and disconnected.

Female failure to achieve was related to male emotional inhibition/overcontrol, and female dependence/incompetence had the strongest relationship with male defectiveness/shame and emotional inhibition/overcontrol. Fear of failure to achieve in females may encourage males to inhibit their emotions in order to maintain stability and support their partners. Males may over-control their emotions to avoid increasing their partner’s fear of failure.

We can observe the frequent appearance of emotional inhibition/overcontrol schemas in males as a complementary schema in females. This highlights its central role in relationship dynamics as a coping strategy, conflict avoidance mechanism and means of emotional stability management. However, while it may provide short-term alleviation, it can also inhibit deep emotional connection and long-term relationship satisfaction. Recognising this may be crucial in working through relationship issues and promoting healthier emotional expression within the relationship. Female feelings of dependency may lead males to feel defective and so control their emotions tightly. To avoid highlighting their partner’s dependency and feelings of incompetence, males may suppress their emotions.

Vulnerability to harm or illness in females was associated with emotional deprivation and mistrust/abuse in males. In addition, subjugation in females found to be related to approval-seeking/recognition-seeking in males. This dynamic may reflect traditional gender roles in which females suppress their needs to protect relationship harmony, while males seek validation through approval-seeking behaviors. This dynamic may contribute to cycles of anger as one partner feels overburdened with emotional caregiving while the other struggles with a sense of inadequacy. Finally, females’ punishment schema was highly related to males’ approval-seeking/recognition-seeking and punishment schemas. Sex differences in maladaptive schemas have been noted in studies by Wegener et al. (2013) and Santos et al. (2018), but these studies did not examine the interaction within schemas in romantic relationships.

### 4.2 Actor-partner dynamics

Emotional deprivation is associated with relationship satisfaction for both sexes, influencing not only individual well-being but also the relationship dynamics between partners. Pilkington et al. (2021) emphasize that emotional deprivation is associated with a feeling of being unloved and unsupported, which correlates with persistent dissatisfaction. The lack of emotional reciprocity can leave both partners unfulfilled and dissatisfied, inhibiting the expression of needs and mutual understanding. Altogether, emotional deprivation affects the basic sense of connection and support, resulting in shared dissatisfaction and heightened negative emotions between partners. This supports the theory that emotional deprivation schema promotes feelings of emotional detachment and unmet needs (Young, 1999), creating emotional defenses that limit intimate communication and the emotional expression of needs.

Our study shows that the abandonment schema is associated with lower relationship satisfaction, particularly for females, which is consistent with the findings of D’Rozario and Pilkington (2021). This may suggest that females may be expressing their anxiety more intensely, affecting relationship dynamics and their partner’s satisfaction, possibly because their feelings of emotional distress are more easily transmitted to their partners compared to males. Similarly, the mistrust/abuse schema appears to be related to relationship satisfaction in a gender differentiated way. Although females may preserve their own satisfaction, their suspiciousness and defensiveness may place a burden on their partners, as also observed by Pilkington et al. (2021). This pattern of behaviour may reflect a potential coping mechanism whereby females reduce their emotional vulnerability by projecting mistrust onto their partners. This dynamic often appears to lead to frustration and emotional exhaustion for male partners. This may suggest that it is more difficult for males to cope with or understand this type of defensive behavior, which may contribute to increased dissatisfaction.

The effect of social isolation schemas on relationship satisfaction is different for each sex. For males, this schema does not influence relationship satisfaction substantially. For females, however, it’s associated with reduced satisfaction without affecting their partner’s satisfaction. This is consistent with research (Young, 1999; Gyesook et al., 2014) highlighting how social isolation can result in differences, relationship avoidance and disconnection, which affects females’ self-esteem and relationship fulfilment. This discrepancy may reflect deeper differences in the emotional needs of both sexes within relationships. Females, who often prioritise social connectedness, may find that social isolation may lowers their self-esteem and feelings of fulfilment, leading to dissatisfaction. On the other hand, males may prioritise other relationship factors, such as autonomy, which may protect them from the negative effects of social isolation on relationship satisfaction. Therefore, the influence of the schema may emphasize the importance of emotional needs in determining relationship satisfaction and suggest that male and female responses to social isolation may differ because of these different priorities. Similarly, the defectiveness/shame schema is associated with females’ personal relationship satisfaction, but has minimal association with males or their partners. Feelings of shame and defectiveness may be linked to trigger behaviors such as withdrawal or seeking reassurance, which are correlated with reduced females’ satisfaction without affecting their partners’ experience in the same way.

The failure and dependence/incompetence schemas do not appear to notably influence relationship satisfaction. However, the schema of vulnerability to harm or illness is particularly associated with reduced males’ relationship satisfaction, with no effect on females, consistent with previous research (Trincas et al., 2014). This schema may contribute to catastrophic beliefs and fears that could influence behaviors and emotions in relationships (Shi et al., 2023). Addressing and changing these schemas might reduce their negative effects and enhance relationship dynamics (Pilkington et al., 2021). Males may be more directly affected by their fears, which is linked to reduced their satisfaction, whereas females may internalise or cope with these fears differently, reducing their impact. The enmeshment/underdeveloped self schema also does not have a major association with relationship satisfaction for either sex, consistent with previous findings (Trincas et al., 2014). This schema centres on overly emotional dependency on others while not compromising one’s individual identity, allowing the person to maintain relationship satisfaction regardless of its presence.

The subjugation schema negatively associated with person’s relationship satisfaction, particularly for females, without affecting their male partners substantially. Research suggests that females who exhibit subjugation schema often experience a decrease in relationship satisfaction, as they may prioritise their partner’s needs over their own, leading to feelings of resentment and emotional neglect (Najafabadi et al., 2021; Eftekhari et al., 2018). Furthermore, Knapík and Slancová (2020) suggest that the self-sacrifice schema does not influence relationship satisfaction. Rather than focusing on the personal costs, individuals tend to emphasise the benefits and positive consequences of their self-sacrificing behavior, such as increased intimacy and partner well-being. The approval-seeking schema negatively associated with person’s relationship satisfaction, particularly for females, without affecting their male partners substantially (Najafabadi et al., 2021). Individuals with approval-seeking often experience stress about acceptance, which may be related to personal dissatisfaction and reduces relationship satisfaction due to feelings of undervaluing. Partners may perceive approval-seeking behaviors positively, but may not be aware of the underlying struggles. Both sexes tend to experience reduced individual satisfaction, creating a cycle of self-focused dissatisfaction. For males, the emotional inhibition schema shows no meaningful association with relationship satisfaction. For females, however, it is associated with a worsening of their satisfaction without any effect on their male partners. Females with emotional inhibition may struggle to suppress feelings, resulting in relationship dissatisfaction. The unrelenting standards schema is strongly associated with lower relationship satisfaction, with a particularly negative impact on male partners (Najafabadi et al., 2021). Females with a high unrelenting standards schema may place stress on themselves and their partners, which may cause dissatisfaction and may leave partners feeling inadequate or criticised.

In terms of negativity, pessimism and punishment schemas and their association with relationship satisfaction, we can see a specific impact on male satisfaction. Males with these schemas tend to be self-critical and focus on the negative in their relationships, which is associated with personal dissatisfaction. This negative perspective may overshadow the positive aspects of the relationship, thereby reducing overall satisfaction. However, this does not appear to have a significant association with partner satisfaction. For females, the presence insufficient self-control schemas within the impaired limits domain is strongly associated with reduced relationship satisfaction. Insufficient self-control may lead to impulsive or irresponsible behavior, further correlating with reduced their relationship satisfaction.

### 4.3 Strengths and limitations

The strength of our research lies in the relatively large samples of healthy couples. Another strength may be that we used the APIM to examine relationship satisfaction by looking at how couples’ schemas interact. However, a limitation of our study is that the depth of analysis is constrained by the lack of availability of related research. Nevertheless, we will continue to explore these relationships in greater depth as more studies in this area emerge. Despite this limitation, the use of APIM is a strength of our research as it allows for a more comprehensive analysis of relationship dynamics. Furthermore, a clear limitation of the present approach is that by focusing exclusively on mixed-sex couples to explore sex differences in relationship satisfaction, the study excludes the experiences of same-sex couples. This limits insight into how early maladaptive schemas and relationship satisfaction play out in different types of romantic relationships. In addition, the nature of the sample may limit the generalisability of the findings to other socio-economic or cultural groups. Another limitation of our study was the relatively short duration of the relationships. The shorter relationship duration in relation to age may indicate that these relationships are either second or subsequent relationships after divorce or widowhood, which may have different dynamics than first long-term relationships. Nevertheless, these limitations, together with the results of the study, suggest the need for further research to better understand the complex factors and their impact on relationship satisfaction.

Future studies could be extended to examine whether patterns of early maladaptive schemas and satisfaction apply to different types of romantic relationships, such as same-sex couples and long-distance partnerships. Future research could benefit from including a wider age range of participants. This could extend from young adulthood to older adulthood. Longitudinal studies could track changes in maladaptive schemas and relationship satisfaction over time.

Further longitudinal studies could examine the development of the relationship between relationship satisfaction and schemas over time, particularly as couples move through life changes, stressors, and milestones. Cross-cultural studies would contribute to a broader perspective on romantic relationships by enriching our understanding of the interplay between cultural norms, the development of relationships and satisfaction outcomes.

Through the exploration of these issues, future research can have a broader insight into the complex dynamics of relationship satisfaction. This has valuable implications for therapy and relationship support interventions.

## 5 Conclusion

Our research improves our understanding of how early maladaptive schemas contribute to relationship outcomes by providing a nuanced understanding of how couples’ schemas interact. Interdependence highlights how the schemas of one partner can complement and reinforce those of the other. We have analysed these dynamics and identified how each partner’s schemas relate to relationship satisfaction, taking into account interactions between partners’ schemas. The emotional deprivation schema is associated with a decrease in satisfaction for both partners. In females, the abandonment schema is associated with a reduction in both their own and their partner’s satisfaction, while the mistrust/abuse schema only affects the male partner. Social isolation and defectiveness/shame schemas lower female satisfaction without affecting their partners, whereas male satisfaction is reduced by vulnerability to harm. Schemas such as failure to achieve, dependence/incompetence and enmeshment have little effect. Approval-seeking schema reduces female satisfaction, and negativity/pessimism is associated with male satisfaction.

Our study relates to improved couple therapy by providing a deeper understanding of how early maladaptive schemas are related to relationship satisfaction.

The focus of therapy should be on the promotion of open communication between partners in order to address unmet emotional needs and promote emotional support. To manage relationship anxiety and promote secure attachment, cognitive behavioral strategies are particularly useful, especially for females. To reduce defensive behaviour and rebuild trust in relationships, confidence-building exercises and schema-focused therapy can help.

Therapy should aim to strengthen social connections and improve communication skills within relationships to address feelings of disconnection, particularly in females. For males, cognitive restructuring and exposure may help to address excessive worry and catastrophizing and promote emotional resiliency. In addition, therapy can help individuals challenge their unrealistic expectations and improve emotional regulation, reducing impulsive behaviors and increasing overall relationship satisfaction.

## Data Availability

The original contributions presented in the study are included in the article/supplementary material, further inquiries can be directed to the corresponding author.
